# Green Coffee Extract Mitigates Fipronil-Induced Endocrine Disruption, Metabolic Disturbances and Oxidative Stress in Male Albino Rats

**DOI:** 10.3390/toxics14050383

**Published:** 2026-04-30

**Authors:** Alaa Hlail Dahham, Mohamed Korish, Samir Mohamed El Rayes, Nadia A. El-Fahla, Ibrahim E. Helal, Heba M. A. Abdelrazek

**Affiliations:** 1 Department of Chemistry, Faculty of Science, Suez Canal University, Ismailia 41522, Egypt; 2 Department of Agriculture, Faculty of Environmental Sciences, King Abdulaziz University, P.O. Box 80208, Jeddah 21589, Saudi Arabia; 3 Department of Zoology, Faculty of Science, Suez Canal University, Ismailia 41522, Egypt; 4 Department of Physiology, Faculty of Veterinary Medicine, Suez Canal University, Ismailia 41522, Egypt

**Keywords:** adipokines, endocrine disruptors, fipronil, green coffee extract, oxidative stress, inflammation, metabolic disorders

## Abstract

This study evaluated the protective effects of green coffee (*Coffea arabica* L.) extract (GCE) against metabolic and endocrine disturbances induced by fipronil (FIP) in male rats. Animals were randomly allocated into four groups (*n* = 6): control, GCE (100 mg/kg), FIP (4.85 mg/kg), and combined FIP + GCE, and treated orally for 90 days. FIP exposure significantly impaired glucose homeostasis, as indicated by a 14.8% increase in the oral glucose tolerance test (OGTT) response and a 2.4-fold increase in the homeostatic model assessment of insulin resistance (HOMA-IR). It also disrupted lipid metabolism, with marked elevations in triglycerides (74.10%) and total cholesterol (57.55%). Endocrine imbalance was evident, including increased resistin levels (113.86%) and reduced triiodothyronine (T3; −37.5%), adiponectin (−42.73%), and high-density lipoprotein (HDL; −9.31%). Oxidative stress and inflammation were significantly enhanced, as demonstrated by elevated malondialdehyde (MDA; +93.56%) and pro-inflammatory cytokines (IL-1β: +246.56%; IL-6: +275%), alongside a reduction in total antioxidant capacity (TAC; −45.24%). Additionally, serum albumin levels decreased markedly (−54%). Co-administration of GCE significantly improved metabolic, hormonal, and inflammatory parameters, including insulin resistance (HOMA-IR). Histopathological analysis further confirmed its protective effects on hepatic and renal tissues. Overall, GCE mitigates FIP-induced metabolic and endocrine dysfunction, likely through its antioxidant and anti-inflammatory properties.

## 1. Introduction

Fipronil (FIP) is a phenylpyrazole insecticide extensively used in agriculture, veterinary medicine, and public health for broad-spectrum pest control. It exhibits higher efficacy than several conventional insecticides, including carbamates, organophosphates, and pyrethroids [1]. The toxicological action of FIP primarily involves inhibition of γ-aminobutyric acid (GABA)-gated chloride channels in the central nervous system, leading to neuronal hyperexcitation and eventual death of target organisms. Despite its effectiveness, increasing concerns have been raised regarding its environmental and health impacts. FIP is classified as a possible human carcinogen (Class C) and is characterized by high environmental persistence [2]. In addition, both FIP and its major metabolite, fipronil-sulfone, are chemically stable and prone to bioaccumulation in soil, water, and the food chain, thereby increasing the risk of chronic exposure [3].

Accumulating evidence indicates that pesticide exposure, including FIP, may adversely affect metabolic and endocrine functions. At low doses, such compounds can act as endocrine-disrupting chemicals (EDCs), interfering with hormonal regulation and contributing to metabolic and reproductive disorders [4]. Experimental and epidemiological studies have reported associations between pesticide exposure and alterations in thyroid function, lipid metabolism, and glucose homeostasis, suggesting a potential role in the development of metabolic syndrome [5].

Metabolic syndrome is a multifactorial condition characterized by insulin resistance, dyslipidemia, central obesity, and impaired glucose tolerance. These abnormalities are strongly associated with oxidative stress and chronic low-grade inflammation, which may be triggered or exacerbated by environmental toxicants such as pesticides [6,7]. Therefore, identifying effective interventions to mitigate pesticide-induced metabolic disturbances is of considerable importance [7,8].

Natural products have recently gained attention as potential protective agents against chemical toxicity. Medicinal plants are rich in bioactive compounds with antioxidant and anti-inflammatory properties [9]. Among these, green coffee (*Coffea arabica* L.) has emerged as a promising nutraceutical. Unlike roasted coffee, green coffee beans retain high levels of chlorogenic acids, which exhibit potent antioxidant, anti-inflammatory, and metabolic regulatory effects [10].

Previous studies have demonstrated that green coffee extract (GCE) improves glucose homeostasis, modulates lipid metabolism, and reduces oxidative stress [11,12]. However, evidence regarding its protective effects against pesticide-induced metabolic and endocrine disruptions, particularly those associated with FIP exposure, remains limited.

Accordingly, the present study aimed to evaluate the protective effects of GCE against FIP-induced metabolic, oxidative, inflammatory, and endocrine alterations in a rat model.

## 2. Materials and Methods

### 2.1. Plant Material and Extraction

Dried green coffee beans were purchased from a local market in Ismailia, Egypt, and processed at the Department of Botany, Faculty of Science, Suez Canal University. The beans were ground into a fine powder, and 200 g was macerated in 1 L of 99% methanol for five days at room temperature with continuous agitation. The solvent was removed under reduced pressure using a rotary evaporator.

The extract was prepared by dissolving 20 g of the dried residue in 2 mL Tween 80 (Sigma-Aldrich, St. Louis, MO, USA) and diluting to 100 mL with distilled water (20% *w*/*v*), as previously described by Adebayo et al. [13]. Phytochemical characterization was performed using HPLC (Agilent Technologies 1260 Infinity series, Waldbronn, Germany) equipped with an Eclipse C_18_ column (4.6 × 250 mm, 5 μm; Agilent Technologies, Santa Clara, CA, USA). The mobile phase consisted of water (A) and 0.05% trifluoroacetic acid in acetonitrile (B), delivered at a flow rate of 0.9 mL/min using a gradient elution program. Detection was carried out at 280 nm, with an injection volume of 5 μL and column temperature maintained at 40 °C. Quantification of phenolic and flavonoid compounds was achieved using external standards and expressed as µg/g extract.

### 2.2. Experimental Animals

Twenty-four male Wistar rats (180–220 g) were obtained from the Faculty of Science, Ain Shams University, Egypt. Animals were housed under standard laboratory conditions (23–25 °C, 50 ± 2% humidity, natural light/dark cycle) with free access to food and water in accordance with internationally recognized animal care guidelines (National Research Council, 2011) [14]. After a 2-week acclimatization period, experimental procedures were conducted in accordance with institutional ethical guidelines (REC 59/2022, Suez Canal University, Ismailia, Egypt, 2022).

### 2.3. Experimental Protocol

Rats were randomly allocated into four groups (*n* = 6 per group):

- Control group received distilled water (vehicle).
- GCE group received a methanolic GCE at a 100 mg/kg BW dose (100 mg/kg body weight/day).
- FIP group received FIP (Zhejiang Yongnong Chem. Co., Wenzhou, China) 10% *w*/*v* in water (4.85 mg/kg body weight/day).
- GCE + FIP group (co-administration, 30 min interval).

All treatments were administered orally by gavage for 90 consecutive days.

The selected FIP dose represents a sub-chronic exposure level widely used to induce metabolic and oxidative disturbances without acute toxicity [15,16,17]. The GCE dose was chosen based on previous studies demonstrating antioxidant and metabolic regulatory effects [18,19,20]. In addition, ethical considerations for animal experimentation were followed in accordance with the 3Rs (Reduction, Refinement, and Replacement) to minimize animal use while maintaining statistical validity. The primary endpoint of this study was the homeostatic model assessment of insulin resistance (HOMA-IR), as a key indicator of insulin resistance and metabolic dysfunction. The sample size was based on prior studies showing a 25–35% difference in HOMA-IR between control and treated groups, indicating a large effect size (Cohen’s d ≈ 1.2–1.5). With an alpha level of 0.05 and 80% power, the minimum required sample size was approximately 5–6 animals per group, so 6 rats per group were used in this study.

The expected variability for HOMA-IR was derived from previously published studies reporting a standard deviation of approximately 0.5–0.8 in similar experimental models [19,20,21]. Sample size estimation was performed assuming a two-group comparison of HOMA-IR, with a significance level (α) of 0.05 and a statistical power of 80%, using G*Power software (version 3.1). A post hoc power analysis based on the observed differences and variability in HOMA-IR confirmed that the achieved statistical power exceeded 80% at α = 0.05.

### 2.4. Serum and Tissue Sampling

At the end of the experimental period, rats were anesthetized, and blood samples were collected via the retro-orbital plexus. Serum was separated and stored at −80 °C for further biochemical analysis. EDTA-treated blood was used for hematological analysis.

Liver, kidneys, and adipose tissues (epididymal and sub-lumbar) were excised, weighed, and processed for histological and molecular analyses. Portions of adipose tissue were stored at −80 °C for gene expression analysis.

### 2.5. Body and Organ Weights

Body weights were recorded at baseline and at the end of the experiment. Relative organ weights were calculated as

relative organ weight (%) = (organ weight/body weight) × 100.

### 2.6. Oral Glucose Tolerance Test (OGTT) and HOMA-IR Calculation

Following overnight fasting (10–12 h), baseline glucose levels were measured. Rats were then administered 40% glucose solution (1 g/kg) [21], and blood glucose levels were recorded at 0–180 min. Serum insulin levels were determined by an ELISA kit (Abnova Co., Heidelberg, Germany). Insulin resistance was estimated using the HOMA-IR index [22]:

HOMA-IR = (fasting insulin × fasting glucose)/405.

### 2.7. Enzyme-Linked Immunosorbent Assay (ELISA)

Rat ELISA specific kits (catalogue numbers KT-59938, KT-59940, and KT-18885 from Kamiya Biomedicacy Co., Seattle, WA, USA) were used to evaluate the levels of free triiodothyronine (T3), tetraiodothyronine (T4), and interleukin-1beta (IL-1β), respectively, in sera. The T3 and T4 ELISA kits had a detection limit of 1 pg/mL. Moreover, the detection limit of IL-1β was 4.2 pg/mL. Serum resistin levels were determined using a rat resistin ELISA kit (catalogue number RD391016200R, Biovendor Co., Brno, Czech Republic). The resistin ELISA kit had a detection limit of 0.05 ng/mL. A rat ELISA kit (R&D Corp., Minneapolis, MN, USA) was used to measure the serum adiponectin level as per the method detailed by Liu et al. [23]. The analyses were conducted following standardized protocols.

### 2.8. Biochemical Parameters Assay

Aspartate aminotransferase (AST) and alanine aminotransferase (ALT) were estimated via the standard spectrophotometric method following the procedures of Duncan et al. [24]. Serum creatinine, uric acid, and urea were estimated spectrophotometrically using an automatic analyzer according to Greenwald’s description of the Jaffe reaction [25]. Total protein (TP) was assessed via the biuret assay [26,27]. The colorimetric methodology was applied to perform the spectrophotometric analysis of serum albumin (Alb) according to Doumas et al. [28]. Bio Diagnostic Co., Cairo, Egypt Kits were used for evaluate high-density lipoprotein (HDL) cholesterol, triglycerides (TG), and total cholesterol (TC) in sera. Oxidative stress indicators malondialdehyde (MDA) and the total antioxidant capacity (TAC), were measured using standard spectrophotometric methods and commercial kits (Labour Diagnostika Nord GmbH & Co. KG, Nordhorn, Germany) according to manufacturer instructions. were followed to quantify.

### 2.9. Histopathology

Formalin-fixed kidney and liver tissue samples from different groups were passed into in 70% and 100% ethyl alcohol after washing with tap water the next day. Specimens were trimmed and cut into 4 μm-thick sections using a slide microtome. The resulting sections were sliced on glass slides, deparaffinized, and subjected to hematoxylin and eosin staining to evaluate the histological alterations on tissue sections under a light microscope [29]. The examination was performed by an investigator who was blinded to the experimental group allocation to minimize observational bias.

### 2.10. Immunohistochemistry

Formalin-fixed paraffin-embedded epididymal fat was used for immunohistochemistry. Deparaffinized sections were hydrated and washed with 0.1 M phosphate-buffered saline (PBS). Endogenous peroxidases were inhibited by incubating the sections in methanol containing hydrogen peroxide, then washing in Tris-buffered saline (TBS). Tissue slices were incubated overnight at 4 °C with an anti– interleukin-6 (IL-6) primary antibody (rabbit polyclonal, 1:200 dilution; Abcam, UK). After rinsing three times, the sections were exposed to a biotinylated secondary antibody and a streptavidin-HRP detection system for 30 min. After further washing, diaminobenzidine tetrahydrochloride (DAB) was added for 5 to 10 min to complete the enzyme reaction. Finally, the slides were counterstained with hematoxylin for accurate tissue identification [30].

Cells with cytoplasmic IL-6 immunoreactivity were analyzed in epididymal adipose tissue sections. Positive staining, indicated by brown deposits, was observed in adipocytes and interstitial cells. A semi-quantitative analysis was performed by an investigator who was blinded to the experimental group allocation to minimize observational bias. Measurement of the integrated optical density (IOD) of the DAB signal was done by using ImageJ software Version 1.53t (ImageJ, Bethesda, MD, USA) following standardized procedures. Mean IOD values were calculated from six randomly selected high-power fields (400×) for each tissue sample to assess IL-6 expression intensity.

### 2.11. Real-Time Polymerase Chain Reaction

Fats were subjected to RNA extraction using the Applied Biotechnology kit with Cat. No. ABT002, Ismailia, Egypt. The obtained mRNA was reverse-transcribed into cDNA using the Applied Biotechnology kit, Cat. No. AMP11, Egypt). Both mRNA extraction and cDNA synthesis were performed according to the manufacturer’s instructions. Quantification of IL-6 expression levels was performed using the StepOne™ Real-Time PCR System with the High ROX kit (Cat. No. AMP04, Applied Biotechnology, Ismailia, Egypt). The following primers were used: Forward: TCCTACCCCAACTTCCAATGCTC and Reverse: TTGGATGGTCTTGGTCCTTAGCC [31]. The thermal cycling conditions were 45 cycles at 95 °C for 20 s, followed by 65 °C for 4–5 s, and finally 72 °C for 8 s. The gathered data were undertaken for normalization against β-actin as a housekeeping gene. β-actin was selected as the housekeeping gene because it is widely used and validated in rat adipose tissue gene expression studies. Preliminary evaluation confirmed stable expression across all experimental groups with minimal Ct variation. Therefore, it was considered suitable for normalization in this experimental model. Finally, the results of the fold expression were obtained using the ΔΔCt method [32]. To validate the use of the ΔΔCt method for relative gene expression analysis, standard curves were generated for both IL-6 and β-actin primer sets using serial dilutions of cDNA. The amplification efficiency of each primer pair was calculated from the slope of the standard curve according to the equation E = (10^(−1/slope)^ − 1) × 100. The amplification efficiencies for IL-6 and β-actin were within the acceptable range of 90–110%, and the correlation coefficients (R^2^) of the standard curves exceeded 0.99, indicating high linearity and reliability of the qPCR assays.

### 2.12. Statistical Analysis

Statistical analyses were conducted using GraphPad Prism (version 5.01, San Diego, CA, USA). Prior to statistical analysis, data normality was assessed using the Shapiro–Wilk test, and homogeneity of variance was evaluated using Levene’s test. For normally distributed datasets, one-way ANOVA followed by Tukey’s post hoc test was applied. When the normality assumption was violated, the Kruskal–Wallis test followed by Dunn’s multiple comparisons test was used. A *p*-value of <0.05 was considered statistically significant.

## 3. Results

### 3.1. HPLC Analysis for Green Coffee Extract

High-performance liquid chromatography (HPLC) analysis demonstrated that chlorogenic acid and pyrocatechol were the predominant constituents of the GCE. Other relatively abundant compounds included naringenin, ferulic acid, catechin, and syringic acid.

**Figure 1:**
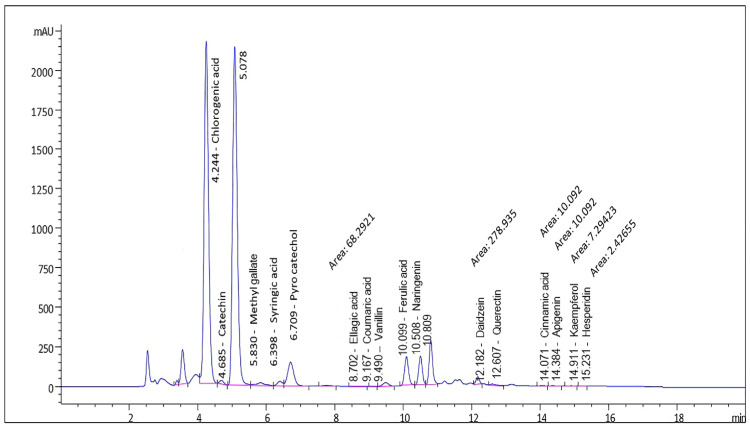
HPLC pattern for the green coffee methanolic extract’s quantitative and qualitative identification components.

Compounds detected at lower concentrations comprised methyl gallate, daidzein, vanillin, gallic acid, rutin, quercetin, and ellagic acid. In addition, kaempferol, apigenin, hesperidin, cinnamic acid, and coumaric acid were identified in trace amounts compared with the aforementioned constituents (Figure 1; Table 1).

### 3.2. Body and Organs Weight

No statistically significant differences (*p* > 0.05) were observed among the experimental groups in terms of initial body weight, final body weight, or absolute and relative kidney weights. In contrast, FIP exposure resulted in a significant increase (*p* ≤ 0.05) in both absolute and relative liver weight, as well as in sub-lumbar and epididymal adipose tissue weights, compared with the control group. Co-administration of GCE with FIP significantly reduced (*p* ≤ 0.05) adipose tissue weights relative to the FIP-treated group. However, liver weight remained elevated, with no statistically significant improvement observed following GCE treatment (Table 2).

### 3.3. HOMA-IR and OGTT Responses

**Figure 2:**
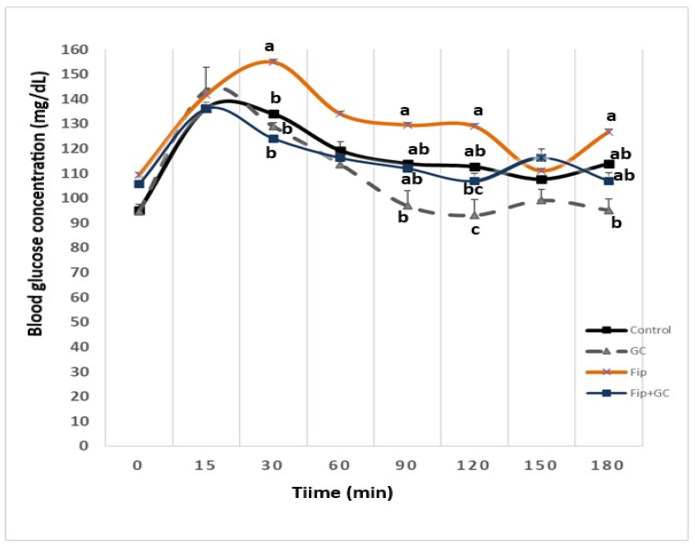
Effect of oral green coffee extract (GCE) 100 mg/kg BW administration on oral glucose tolerance test (OGTT) in FIP (4.85 mg/kg BW) intoxicated male Wistar rats (*n* = 24) for ninety days (mean ± SD). Superscript letters (a, b, and c) among different treatment groups refer to significance at (*p* ≤ 0.05).

As the primary endpoint, HOMA-IR increased significantly following FIP exposure compared with control rats, indicating marked insulin resistance. FIP-treated rats exhibited significantly elevated (*p* ≤ 0.05) blood glucose levels at 30, 90, 120, and 180 min during the OGTT compared with control animals. Co-treatment with GCE significantly attenuated (*p* ≤ 0.05) glucose elevations compared with the FIP-only group (Figure 2). Consistent with these findings, HOMA-IR values indicated a significant increase (*p* ≤ 0.05) in insulin resistance following 90 days of FIP exposure. This effect was significantly mitigated (*p* ≤ 0.05) by co-administration with GCE (Table 3).

### 3.4. Resistin, Adiponectin, and Thyroid Hormones

Chronic FIP exposure led to a significant decrease (*p* ≤ 0.05) in serum T3 levels compared with control animals. Co-treatment with GCE significantly restored (*p* ≤ 0.05) T3 concentrations relative to the FIP group. No statistically significant differences were detected in T4 levels among the experimental groups. FIP administration significantly increased (*p* ≤ 0.05) serum resistin levels and decreased (*p* ≤ 0.05) adiponectin levels compared with controls. These alterations were significantly reversed (*p* ≤ 0.05) in rats receiving combined FIP and GCE treatment, as evidenced by reduced resistin and elevated adiponectin levels relative to the FIP group (Table 3).

### 3.5. Biochemical Parameters

Exposure to FIP resulted in significant increases (*p* ≤ 0.05) in serum levels of AST, ALT, creatinine, urea, uric acid, IL-1β, MDA, TG, and TC, compared with the control group. Conversely, significant reductions (*p* ≤ 0.05) were observed in TAC, HDL, TP, and Alb. Co-administration of GCE significantly ameliorated (*p* ≤ 0.05) these biochemical disturbances. Specifically, GCE reduced markers of hepatic and renal dysfunction, inflammation, oxidative stress, and dyslipidemia; while restoring TAC, HDL, TP, and Alb levels compared with the FIP-treated group (Table 4).

### 3.6. Histopathology

**Figure 3:**
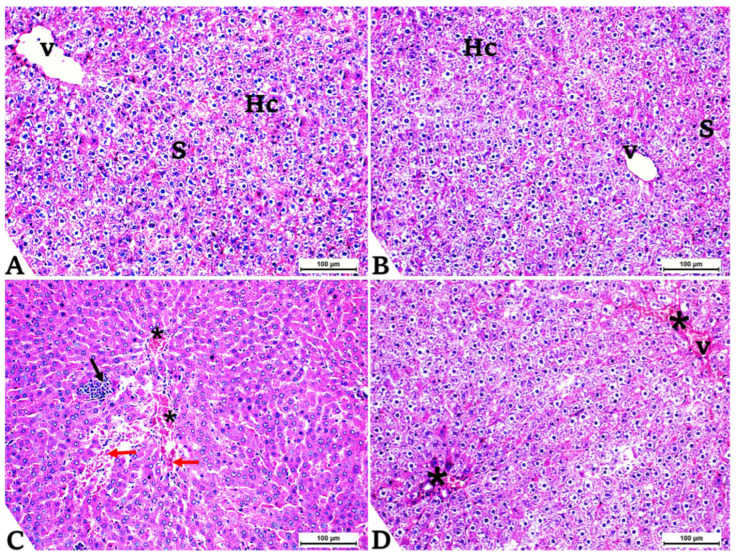
Histological evaluation of liver sections (H&E staining, ×200, bars 100 μm). (**A**,**B**) are representative sections from the control group and green coffee methanolic extract (GCE) 100 mg/kg BW supplemented group, respectively, show normal liver histology, including normal hepatocytes (Hc) with distinct nuclei (N) and weakly stained cytoplasm, central veins (v), and sinusoids (S). (**C**) representative section from the fipronil (FIP)-treated (4.85 mg/kg BW) group shows changes such as dilatation and congestion of the central veins and sinusoids (*), hemorrhage (red arrows), and inflammation (black arrows). (**D**) Representative section from the fipronil and green coffee methanolic extract (FIP + GCE) co-administered group shows significant improvement in liver tissue, * indicates dilation and congestion of the central veins and sinusoids.

The examination showed a normal appearance in the hepatic tissues of the control group (Figure 3A) and the GCE-supplemented group (Figure 3B). The liver parenchyma displayed a normal arrangement of hepatocytes containing central veins and scattered sinusoids. The treated livers with FIP exhibited histological changes, including dilation and congestion of the central vein and sinusoids, parenchymal hemorrhage, and inflammation (Figure 3C). The administration of GCE to the FIP group markedly improved the hepatic tissue (Figure 3D).

**Figure 4:**
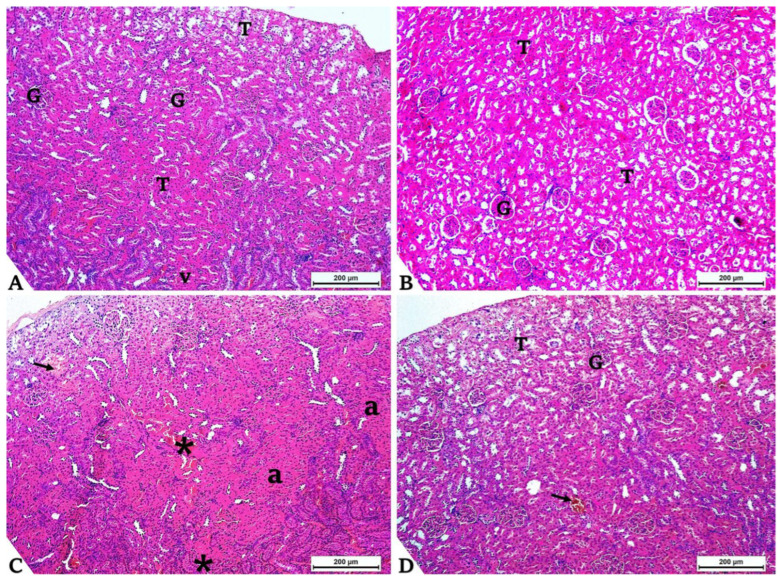
Histological examinations of hematoxylin–eosin-stained kidney sections (H&E × 100, Bar 200 μm), Abbs; T, renal tubules; G, glomerulus; v, renal vein. (**A**) Control and green coffee methanolic extract (GCE) 100 mg/kg BW supplemented (**B**) groups exhibit normal kidney histology. (**C**) Representative section from the fipronil (FIP)-treated (4.85 mg/kg BW) group shows changes included atrophy of renal tubules (a), congestion of the renal vein (*), and hemosiderosis (black arrow). The (**D**) representative section from the fipronil and green coffee methanolic extract group (FIP + GCE) co-administered group shows significant improvement in renal tissues, with lesions still present, black arrow indicates hemosiderosis.

Kidney slices from control rats (Figure 4A) and those from the GCE-treated group (Figure 4B) exhibited a typical histological structure of renal parenchyma with no pathological lesions. Meanwhile, renal sections from rats treated with FIP (Figure 4C) showed numerous alterations, including vascular congestion, hemorrhage, tubular atrophy, and hemosiderosis. The co-administration of GCE with FIP enhanced the appearance of renal tissue (Figure 4D).

### 3.7. Immunohistochemical Expression of IL-6

**Figure 5:**
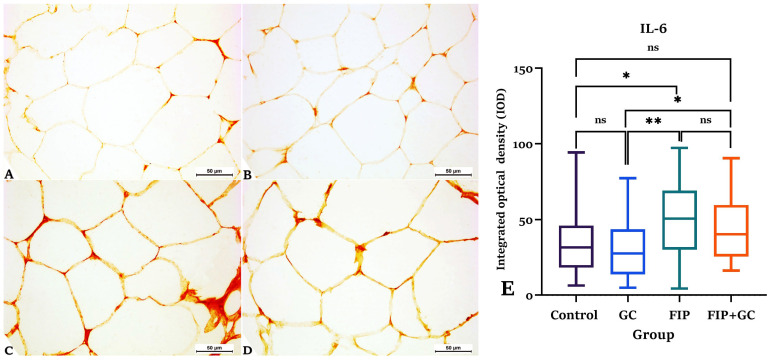
Immunohistochemical analysis indicates the localization of interleukin-6 (IL-6) protein in the epididymal adipose tissues from the control group (**A**), green coffee methanolic extract (GCE) 100 mg/kg BW supplemented group (**B**), fipronil (FIP)-treated (4.85 mg/kg BW) group (**C**), and the fipronil and green coffee methanolic extract (FIP + GCE) co-administered group (**D**), ×400, Bar 50 μm. Strong positive staining with anti-IL-6 antibody is seen within the adipocytes’ membrane and interstitial cells within the adipose tissue from FIP-intoxicated male *Wistar* rats. (**E**) IL-6 immunostaining was quantitatively analyzed as integrated optical density (IOD; mean ± SE). A box-and-whisker plot compares the control, GCE, FIP, and FIP + GCE groups. Statistical analysis used the Kruskal–Wallis test with Dunn’s multiple comparisons (* *p* < 0.05, ** *p* < 0.01, ns = not significant).

Immunohistochemical analysis of epididymal adipose tissue revealed varying IL-6 expression among the groups. The control group (Figure 5A; 68.40 ± 4.90, *p* > 0.05) and GCE group (Figure 5B; 60.20 ± 4.60, *p* > 0.05) had weak IL-6 immunostaining. In disparity, the FIP group (Figure 5C) displayed a significant increase in IL-6 immunostaining (95.50 ± 5.20), especially along adipocyte membranes, matched to the control (*p* = 0.0233) and GCE groups (*p* = 0.0013). The FIP + GCE group (Figure 5D) showed moderate IL-6 staining (86.00 ± 5.00), significantly higher than GCE (*p* = 0.0353) but not significantly different from control or FIP (*p* > 0.05). Quantitative analysis of the IOD values was illustrated in Figure 5.

### 3.8. IL-6 mRNA Expression

**Figure 6:**
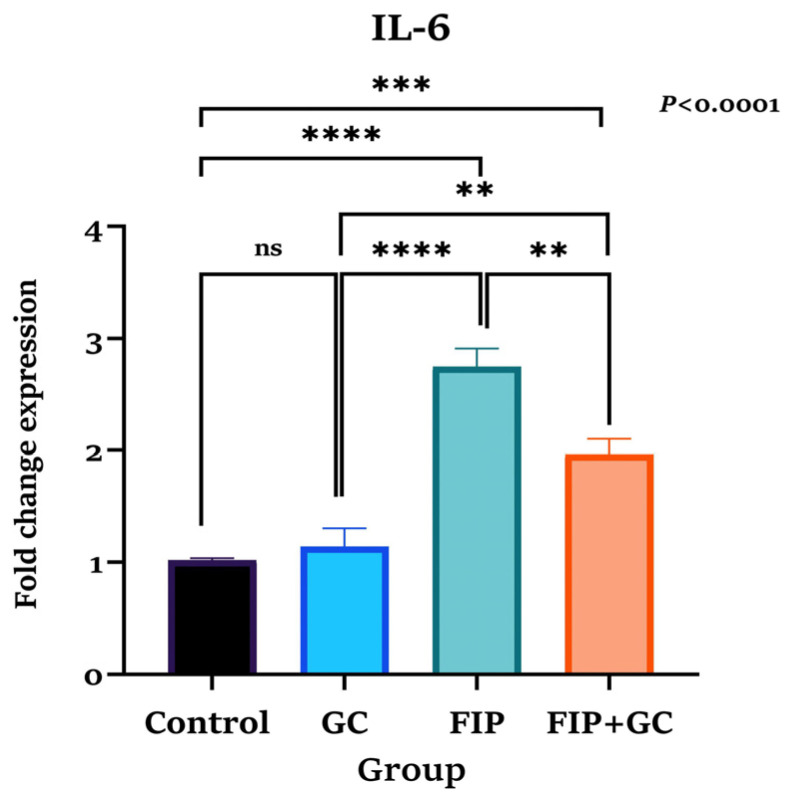
Fold change expression of IL-6 in control, green coffee methanolic extract (GCE) 100 mg/kg supplemented group, FIP (4.85 mg/kg BW) intoxicated, and fipronil and green coffee methanolic extract (FIP + GCE) co-administered male *Wistar* rats (*n* = 24) for ninety days. Data are presented as mean ± SE. Statistical analysis was performed using one-way ANOVA and Tukey’s post hoc tests (** *p* < 0.01, *** *p* < 0.001, **** *p* < 0.0001, ns = not significant).

As shown in Figure 6, IL-6 mRNA expression did not differ significantly between the control (1.02 ± 0.02) and GCE groups (1.14 ± 0.16; *p* = 0.9159). In contrast, FIP exposure resulted in a marked upregulation of IL-6 expression (2.75 ± 0.16; *p* < 0.0001) compared with both groups. Co-administration of GCE significantly reduced IL-6 expression (1.97 ± 0.19; *p* = 0.0026) relative to the FIP-treated group. However, IL-6 expression remained significantly elevated compared with the control and GCE groups (*p* ≤ 0.05).

## 4. Discussion

The present study demonstrated that GCE is a rich source of phenolic compounds, with chlorogenic acids as the predominant constituents, along with flavonoids such as catechin, naringenin, and quercetin, as well as minor polyphenolic components. These bioactive compounds are widely recognized for their antioxidant, anti-inflammatory, and metabolic regulatory properties [33,34]. It is important to note that the phytochemical profile of GCE may vary depending on factors such as coffee variety, geographical origin, and extraction methodology. Despite this variability, chlorogenic acid consistently emerges as the major component, in agreement with previous reports [34].

In the current study, FIP exposure did not significantly affect total body weight; however, it induced a significant increase in liver weight and adipose tissue mass, particularly in sub-lumbar and epididymal fat depots. This pattern suggests a redistribution of lipid storage rather than a generalized increase in body mass. Similar observations have been reported previously [16,17,35], indicating that FIP may disrupt lipid metabolism and promote lipid accumulation. The concurrent elevation in serum TG and total TC further supports this interpretation [36,37]. Mechanistically, these alterations may be attributed to FIP-induced oxidative stress and inflammatory responses, which impair adipocyte function and favor lipid deposition [38].

Notably, co-administration of GCE significantly reduced adipose tissue accumulation and partially normalized organ weights. These findings are consistent with earlier studies demonstrating that GCE enhances lipid oxidation, improves glucose utilization, and reduces visceral fat deposition [19,39,40]. Additionally, GCE may modulate energy balance by influencing appetite regulation and increasing energy expenditure, thereby contributing to reduced fat accumulation [41].

Fipronil exposure also resulted in impaired glucose tolerance, as evidenced by elevated glucose levels during the OGTT, in agreement with previous studies [42]. This impairment may reflect pancreatic β-cell dysfunction and reduced insulin sensitivity [43]. Furthermore, oxidative stress and inflammation induced by FIP are known to disrupt glucose homeostasis [3]. The observed increase in HOMA-IR further confirms the development of insulin resistance, likely mediated through alterations in insulin signaling pathways secondary to oxidative and inflammatory stress [3,44].

Importantly, GCE supplementation significantly improved glucose tolerance and reduced HOMA-IR values. These effects are likely mediated by chlorogenic acids, which have been shown to enhance insulin sensitivity, inhibit hepatic gluconeogenesis, and promote peripheral glucose uptake [45,46]. In addition, GCE may delay intestinal glucose absorption, contributing to improved glycemic control [47].

With respect to endocrine function, FIP exposure led to a significant reduction in T3 levels without affecting T4, suggesting disruption of peripheral thyroid hormone metabolism. Comparable findings have been reported in previous studies [48]. This effect may be linked to oxidative stress, which can impair thyroid peroxidase activity and deiodinase enzymes responsible for hormone synthesis and conversion [49].

The GCE co-treatment significantly restored T3 levels, likely through its antioxidant properties, which may preserve the activity of enzymes involved in thyroid hormone metabolism. Chlorogenic acid, in particular, may play a key role in this protective effect [46,50,51].

The FIP exposure also disrupted adipokine balance, as indicated by increased resistin and decreased adiponectin levels. Elevated resistin is associated with insulin resistance and pro-inflammatory signaling [52,53], whereas reduced adiponectin is linked to impaired glucose regulation and enhanced lipid accumulation. These alterations are consistent with adipose tissue dysfunction induced by oxidative stress and endocrine-disrupting effects of FIP [54,55].

Conversely, GCE supplementation effectively improved adipokine profiles by reducing resistin and increasing adiponectin levels. This modulatory effect may be attributed to the attenuation of oxidative stress and inflammatory pathways, leading to improved adipose tissue function and metabolic homeostasis [19,56,57].

Biochemically, FIP exposure resulted in elevated hepatic enzymes (ALT and AST), renal function markers (urea, creatinine, and uric acid), and lipid parameters (TG and TC), indicating hepatotoxicity, nephrotoxicity, and dyslipidemia. These findings are consistent with previous reports [38,58,59,60] and are likely mediated by oxidative stress and inflammation, which compromise cellular integrity and metabolic processes [38]. The observed reductions in Alb, TP, HDL, and TAC further reflect impaired liver function and weakened antioxidant defenses [39,58,59].

The observed reductions in Alb, TP, HDL, and TAC further reflect impaired liver function and weakened antioxidant defenses [60,61,62]. Additionally, GCE favorably modulated lipid metabolism by lowering TG levels and increasing HDL concentrations [19,63].

A central finding of this study is the induction of oxidative stress and inflammation following FIP exposure, as evidenced by increased MDA and IL-1β, along with decreased TAC. These results are consistent with previous studies demonstrating that FIP enhances reactive oxygen species (ROS) generation and activates pro-inflammatory pathways, including nuclear factor kappa B (NF-κB) signaling [3,64,65].

Mitochondrial dysfunction may further amplify these effects by promoting ROS production and releasing damage-associated molecular patterns (DAMPs), which trigger inflammatory responses [66,67]. Elevated IL-1β levels indicate activation of inflammatory cascades that contribute to metabolic dysregulation and tissue injury [68,69].

Importantly, GCE markedly attenuated oxidative stress and inflammation, as demonstrated by reduced MDA and IL-1β levels and restoration of TAC. These effects may involve inhibition of NF-κB signaling and suppression of inflammasome activation, particularly the NLRP3 pathway, mediated by chlorogenic acid and related polyphenols [49,70]. Furthermore, GCE may regulate immune responses by limiting the production of pro-inflammatory mediators [71,72].

Collectively, these findings suggest that FIP induces metabolic disturbances through a multifactorial mechanism involving oxidative stress, inflammation, endocrine disruption, and impaired metabolic signaling. Oxidative stress appears to play a central role, linking these pathological processes.

In contrast, GCE exerts a broad protective effect by targeting these interconnected pathways, thereby restoring metabolic balance. These results highlight the potential of GCE as a natural therapeutic strategy for mitigating pesticide-induced metabolic dysfunction.

## 5. Conclusions

Chronic oral exposure to FIP-induced marked metabolic and endocrine disturbances, including impaired glucose tolerance, thyroid dysfunction, dysregulated adipokine secretion, and altered lipid metabolism. These alterations were closely associated with increased oxidative stress and inflammation, as evidenced by enhanced lipid peroxidation, elevated pro-inflammatory cytokines, and reduced antioxidant capacity. Co-administration of GCE effectively mitigated these adverse effects, leading to improvements in glycemic control, lipid profile, thyroid hormone balance, and adipokine regulation. The protective effects of GCE are likely attributable to its high content of bioactive phytochemicals, particularly phenolic compounds such as chlorogenic acid, which exhibit potent antioxidant and anti-inflammatory activities.

Importantly, HOMA-IR, defined a priori as the primary endpoint of this study, was significantly increased following FIP exposure and was markedly improved by GCE treatment, confirming its central role in the metabolic effects observed.

Collectively, these findings support the potential of GCE as a natural intervention to counteract FIP-induced metabolic dysfunction. Several limitations should be acknowledged. First, the use of an animal model restricts the direct translation of these findings to humans; therefore, well-designed clinical and translational studies are required to confirm efficacy and safety in human populations. Second, although the results suggest involvement of oxidative stress and inflammatory pathways, key molecular mechanisms were not directly investigated. In particular, signaling pathways such as nuclear factor erythroid 2–related factor 2 (Nrf2), NF-κB, and insulin signaling cascades were not assessed, nor were protein-level validations performed. Additionally, the absence of thyroid-stimulating hormone (TSH) measurement limits comprehensive evaluation of the hypothalamic–pituitary–thyroid axis function. Future studies should incorporate TSH alongside T3 and T4 to better characterize endocrine disruption. Finally, standardization of GCE composition and identification of its active constituents are essential for improving reproducibility and facilitating its potential therapeutic application.

## Figures and Tables

**Table 1:** HPLC analysis results for the ingredient in the green coffee methanolic extract sample: the multi-wavelength chromatogram of the green coffee methanolic extract for quantitative and qualitative identification was monitored at 280 nm.

Compound	Concentration (µg/g)
Chlorogenic acid	131,904.12
Pyrocatechol	13,838.13
Naringenin	7976.91
Ferulic acid	4994.71
Catechin	2329.09
Syringic acid	1077.23
Methyl gallate	877.29
Daidzein	870.56
Vanillin	553.70
Gallic acid	517.01
Rutin	403.05
Quercetin	282.64
Ellagic acid	119.71
Kaempferol	38.82
Apigenin	28.10
Hesperetin	7.19
Cinnamic acid	5.15
p-Coumaric acid	3.57

**Table 2:** Body and organs (absolute and relative) weight of male *Wistar albino* rats administered green coffee methanolic extract (GCE) 100 mg/kg. BW and fipronil (FIP) 4.85 mg/kg BW for ninety continuous days.

	Control	GCE	FIP	FIP + GCE
Initial BW (g)	169.00 ± 11.49	166.00 ± 10.80	175.00 ± 6.83	166.75 ± 9.07
C.V. (%)	6.80	6.51	3.90	5.44
Final BW (g)	321.75 ± 26.13	336.00 ± 30.95	407.00 ± 66.11	345.00 ± 32.87
C.V. (%)	8.12	9.21	16.24	9.53
Absolute liver weight (g)	10.10 ± 1.34 ^b^	10.03 ± 1.44 ^b^	14.94 ± 2.54 ^a^	12.50 ± 1.15 ^ab^
C.V. (%)	13.2	14.36	17.00	9.2
Relative liver weight (%)	3.14 ± 0.31 ^ab^	2.98 ± 0.23 ^b^	3.67 ± 0.33 ^a^	3.52 ± 0.09 ^a^
C.V. (%)	9.87	7.72	8.99	2.56
Absolute kidney weight (g)	1.02 ± 0.16	0.97 ± 0.12	0.97 ± 0.31	1.10 ± 0.11
C.V. (%)	15.69	12.37	31.96	10.00
Relative kidney weight (%)	0.32 ± 0.05	0.29 ± 0.02	0.23 ± 0.06	0.31 ± 0.04
C.V. (%)	15.63	8.70	26.09	12.90
Absolute epididymal weight (g)	3.48 ± 0.51 ^b^	2.16 ± 0.38 ^b^	11.57 ± 1.84 ^a^	3.57 ± 0.97 ^b^
C.V. (%)	14.66	17.59	15.90	27.17
Relative epididymal weight (%)	1.08 ± 0.13 ^b^	0.65 ± 0.12 ^b^	2.90 ± 0.68 ^a^	1.04 ± 0.30 ^b^
C.V. (%)	12.04	18.46	23.45	28.85
Absolute sublumbar weight (g)	4.75 ± 1.57 ^b^	3.74 ± 0.43 ^b^	9.75 ± 2.71 ^a^	5.11 ± 1.15 ^b^
C.V. (%)	89.71	11.50	27.79	22.50
Relative sublumbar weight (%)	1.62 ± 0.61 ^ab^	1.43 ± 0.26 ^b^	2.39 ± 0.54 ^a^	1.09 ± 0.35 ^b^
C.V. (%)	37.65	18.18	22.59	32.11

Superscript letters (a and b) within the same row refer to significance at (*p* ≤ 0.05). Data is represented as mean ± SD. Abbs; BW, body weight; C.V., coefficient of variation.

**Table 3:** Effect of chronic exposure of green coffee extract (GCE) 100 mg/kg BW on the thyroid hormones, adiponectin and resistin in intoxicated rats with fipronil (FIP) 4.85 mg/kg BW for ninety continuous days.

	Control	GCE	FIP	FIP + GCE
HOMA-IR	0.27 ± 0.02	0.25 ± 0.01 ^c^	0.36 ± 0.02 ^a^	0.31 ± 0.01 ^b^
C.V. (%)	7.41	4	5.56	3.23
T3 (ng/dL)	2.56 ± 0.01 ^a^	2.56 ± 0.02 ^a^	1.60 ± 0.01 ^c^	2.27 ± 0.01 ^b^
C.V. (%)	0.39	0.78	0.63	0.44
T4 (μg/dL)	4.17 + 0.03	4.17 + 0.01	4.17 + 0.05	4.17 + 0.06
C.V. (%)	0.72	0.24	1.20	1.84
Resistin (ng/mL)	3.32 ± 0.08 ^c^	3.30 ± 0.01 ^c^	7.10 ± 0.04 ^a^	4.95 ± 0.10 ^b^
C.V. (%)	2.41	3.03	0.56	2.02
Adiponectin (μg/mL)	8.87 ± 0.01 ^a^	8.83 ± 0.01 ^a^	5.08 ± 0.03 ^c^	7.16 ± 0.08 ^b^
C.V. (%)	0.11	1.13	0.59	1.12

Superscript letters (a, b, and c) within the same row refer to significance at (*p* ≤ 0.05). Data is represented as mean ± SD. Abbs; HOMA-IR, Homeostasis Model Assessment of Insulin Resistance; T3, Triiodothyronine (ng/dL); T4, Thyroxine (μg/dL); C.V., Coefficient of variation.

**Table 4:** Effect of chronic exposure of green coffee extract (GCE) 100 mg/kg BW on the biochemical parameters in the intoxicated rats with fipronil (FIP) 4.85 mg/kg BW for ninety continuous days.

	Control	GCE	FIP	FIP + GCE
AST (U/L)	91.75 ± 0.29 ^c^	89.15 ± 0.45 ^d^	160.40 ± 0.65 ^a^	101.45 ± 1.02 ^b^
C.V. (%)	0.32	0.51	0.41	1.01
ALT (U/L)	24.15 ± 0.20 ^c^	24.50 ± 0.49 ^c^	44.60 ± 0.57 ^a^	29.30 ± 0.98 ^b^
C.V. (%)	0.83	2	1.28	3.34
TP (g/dL)	6.19 ± 0.03 ^b^	6.25 ± 0.03 ^a^	5.08 ± 0.02 ^d^	5.27 ± 0.03 ^c^
C.V. (%)	0.48	0.48	0.39	0.57
Alb (g/dL)	4.21 ± 0.03 ^a^	4.23 ± 0.02 ^a^	3.67 ± 0.03 ^b^	4.22 ± 0.02 ^a^
C.V. (%)	0.71	0.47	0.82	0.47
TC (mg/dL)	54.88 ± 0.41 ^c^	54.75 ± 0.67 ^c^	89.47 ± 1.51 ^a^	70.74 ± 1.02 ^b^
C.V. (%)	0.75	1.22	1.69	1.44
TG (mg/dL)	62.45 ± 0.45 ^c^	62.08 ± 0.91 ^c^	108.72 ± 1.25 ^a^	88.55 ± 1.34 ^b^
C.V. (%)	0.72	1.47	1.15	1.51
HDL (mg/dL)	15.68 ± 0.29 ^c^	22.64 ± 0.30 ^a^	14.22 ± 0.32 ^c^	19.16 ± 0.41 ^b^
C.V. (%)	1.85	1.33	2.25	2.14
Urea (mg/L)	15.21 ± 0.04 ^c^	15.09 ± 0.02 ^c^	27.68 ± 0.11 ^a^	20.04 ± 0.19 ^b^
C.V. (%)	0.26	0.13	0.40	0.95
Creatinine (mg/L)	0.43 ± 0.01 ^c^	0.41 ± 0.01 ^c^	1.01 ± 0.05 ^a^	0.57 ± 0.01 ^b^
C.V. (%)	2.33	2.44	4.95	1.75
Uric Acid (mg/L)	2.23 ± 0.05 ^c^	2.27 ± 0.01 ^c^	4.10 ± 0.02 ^a^	2.55 ± 0.03 ^b^
C.V. (%)	2.24	0.44	0.49	1.18
IL-1β (pg/mL)	3.93 ± 0.03 ^c^	3.82 ± 0.02 ^c^	13.62 ± 0.13 ^a^	8.77 ± 0.07 ^b^
C.V. (%)	0.76	0.52	0.95	0.80
TAC (mM/L)	1.68 ± 0.02 ^b^	1.81 ± 0.04 ^a^	0.92 ± 0.01 ^d^	1.53 ± 0.01 ^c^
C.V. (%)	1.19	2.21	1.09	0.65
MDA (nmoL/mg)	1.71 ± 0.01 ^c^	1.68 ± 0.02 ^c^	3.31 ± 0.03 ^a^	2.00 ± 0.02 ^b^
C.V. (%)	0.58	1.19	0.91	1.00

Superscript letters (a, b, c, and d) within the same row refer to significance at (*p* ≤ 0.05). Data is represented as mean ± SD. Abbs; AST, Aspartate Aminotransferase; ALT, Alanine Aminotransferase; TP, Total Protein; Alb, Albumin; TC, Total Cholesterol; TG, Triglycerides; HDL, High-Density Lipoprotein; IL-1β, Interleukin-1β; TAC, Total Antioxidant Capacity; MDA, Malondialdehyde; C.V. (%), Coefficient of Variation.

## Data Availability

The original contributions presented in this study are included in the article. Further inquiries can be directed to the corresponding author.

## Abbreviations

The following abbreviations are used in this manuscript:

FIP	Fipronil
GCE	Green Coffee Extract
EDCs	Endocrine-Disrupting Chemicals
OGTT	Oral Glucose Tolerance Test
HOMA-IR	Homeostasis Model Assessment-Estimated Insulin Resistance
ELISA	Enzyme-Linked Immunosorbent Assay
T3	Triiodothyronine
T4	Tetraiodothyronine
ALT	Alanine Aminotransferase
AST	Aspartate Aminotransferase
TP	Total Protein
Alb	Albumin
HDL	High-Density Lipoprotein Cholesterol
TG	Triglycerides
TC	Total Cholesterol
MDA	Malondialdehyde
TAC	Total Antioxidant Capacity
TBS	Tris Buffer Saline
PBS	Phosphate-Buffered Saline
DAB	Diaminobenzidine Tetrahydrochloride
IOD	Integrated Optical Density
NF-κB	Nuclear factor kappa B
ROS	Reactive oxygen species
DAMPs	Damage-associated molecular patterns
TSH	Thyroid-stimulating hormone
